# High-Level Expression of Functionally Active Dengue-2 Non-Structural Antigen 1 Production in *Escherichia coli*

**DOI:** 10.1155/2013/343195

**Published:** 2013-09-08

**Authors:** S. Gowri Sankar, K. J. Dhanajeyan, R. Paramasivan, V. Thenmozhi, B. K. Tyagi, S. John Vennison

**Affiliations:** ^1^Department of Biotechnology, Anna University, BIT Campus, Tiruchirappalli 620 024, India; ^2^Centre for Research in Medical Entomology (CRME) (WHO Collaborating Centre for Lymphatic Filariasis and Dengue), Indian Council of Medical Research, Chinna Chokkikulam, Madurai 625 002, India

## Abstract

Detection of nonstructural protein (NS1) is an important diagnostic marker during acute phase of dengue infection. Not only for diagnostic purpose, the protein had important role in vaccine design as well, as a candidate for studying virus assembly and maturation. Various researchers employed different expression systems and strategies for recombinant NS1 protein production. Attempts to express NS1 protein in prokaryotic and yeast expression system result in formation of insoluble protein which needs to undergo refolding to attain native structural and functional forms. Here, we report the production of soluble NS1 protein in *E. coli* by using appropriate vector and employing suitable culture conditions to maximize protein production. Proteins were purified using metal affinity chromatography. SDS-PAGE and western blot analysis reveal the native structure of NS1 protein. Solid phase ELISA using the recombinantly expressed antigen with positive and negative dengue samples showed that the expressed protein retains its antigenic and immunological properties. To our knowledge, this is the first report on the successful production of functionally active recombinant dengue-2 NS1 protein production without undergoing any *in vitro* posttranslational modification process.

## 1. Introduction

Dengue is an important flavivirus infection which affects millions of people worldwide, particularly in urban and semiurban areas of tropical and subtropical regions. Around 2.5 billion people are in risk zone [1]. Dengue virus is a positive strand RNA virus whose RNA genome is approximately 11 kb in length. It has four antigenically distinct serotypes (dengue virus 1–4). Though dengue infection occurs as a mild febrile, self-limiting illness, that is, dengue fever (DF), its severe forms dengue hemorrhagic fever (DHF) and dengue shock syndrome (DSS) are important public health problem because of its disease burden and high mortality rate [2]. The viral RNA is encoding three structural proteins (nucleocapsid/core protein (C), membrane protein (M), and envelope protein (E)) and seven nonstructural proteins (NS1, NS2A, NS2B, NS3, NS4A, NS4B, and NS5) [3]. 

Dengue nonstructural glycoprotein-1 (NS1) is a glycoprotein of approximately 46 kDa in size. The functional role played by NS1 of dengue virus has not been found out. Besides its confirmed role in viral RNA replication [4], NS1 involvement in assembly and maturation of virus is not conclusive. In the infected cells (mosquito and mammalian cells) the protein exists in different forms like monomer, dimer, and tetramer in different regions [5]. NS1 glycoprotein is anchored in the lumen of endoplasmic reticulum through signal peptidase is cotranslationally modified by host cell signalase. Dengue NS1 protein has been widely studied for its antigenicity as well as its role in promoting secondary dengue infections [6]. Persons suffering from DHF/DSS have antiplatelet and antiendothelial cell antibodies which cross-react with anti-NS1 antibodies [7]. Researchers also found that dengue could be diagnosed even at an early phase by detecting the circulating NS1 antigen [5] or NS1 IgM antibodies [8] during acute phase as conventional ELISA techniques cannot be effectively used. 

Most attempts to express dengue NS1 in *E. coli* and yeast expression system have ended up with insoluble protein. Commonly, the insoluble protein aggregates are biologically less active and usually give low yield, and moreover the process is more time consuming since refolding has to be optimized every time [9]. In view of these drawbacks associated with insoluble protein production in *E. coli*, an attempt was made to express recombinant NS1 production in soluble form. By choosing an appropriate vector system that helps for high-level soluble recombinant protein production, major obstacles associated with heterologous protein expression in *E. coli* have been overcome. Results of SDS-PAGE, western blotting with anti-NS1 monoclonal antibody, and solid phase ELISA have concluded that the expressed recombinant NS1 has retained its antigenicity and biological properties.

## 2. Materials and Methods

Dengue virus-2 (NGC) strain was inoculated into the monolayers of C6/36 cells maintained in Eagle MEM medium supplemented with 15% fetal bovine serum (Invitrogen). After incubation for 72 h, the virus-infected cell culture fluid was harvested, and viral RNA was extracted using viral RNA miniprep kit (Qiagen) according to manufacturer's instruction. 

### 2.1. Reverse Transcriptase Polymerase Chain Reaction

The cDNA encoding the nonstructural gene 1 (NS1) was amplified using the primer pairs NS1-F (5′-CACCATTCAGGCTGTGACCAAGGAGTTGAC-3′) and NS1-R (5′-ATTCGGATAGTGGTTGCGTTGTGAGC-3′) designed according to vector specifications. Regions corresponding to NS1 antigen were amplified using one-step RT-PCR kit (ROCHE). 

Briefly, 5 *μ*L of template RNA was mixed in a total volume of 25 *μ*L of master mix containing 0.5 *μ*L of dNTPs, 1.25 *μ*L of DTTs, 5 *μ*L of RT enzyme buffer, 1 *μ*L each of forward and reverse primer, and 0.5 *μ*L of RT enzyme mix. The viral RNA was reverse-transcribed in a master cycler (Eppendorf, Germany) with the following cycle conditions: reverse transcription at 42°C for 45 min, followed by denaturation at 94°C for 5 mins, 94°C for 60 sec, 58°C for 60 sec, and 72°C for 60 sec with final extension at 72°C for 10 min. The PCR product was resolved on 1% agarose and purified using QIAquick gel extraction kit (Qiagen). The PCR product was sequenced using dideoxy-chain termination method and directly used in cloning. 

### 2.2. Cloning and Expression of NS1 Gene

Two microlitres of the purified PCR product was used in cloning experiment. The PCR product was ligated into pBAD/Thio-TOPO vector (Invitrogen) as per manufacturer's instruction. The ligated product was transformed into Top 10 *E. coli* cells using chemical competent methods, and the transformants were plated on LB agar plates containing 100 *μ*g/mL of ampicillin. Positive transformants were selected and confirmed by colony PCR using both insert- and vector-specific primers and restriction mapping.

### 2.3. Expression and Optimization of Recombinant NS1 Protein

Transformants selected through PCR were further grown in LB medium at 30°C until mid-log phase. Protein expression was induced with different concentrations of arabinose at different temperatures. One mL of medium was drawn every hour to determine optimal inducer concentration for high-level protein expression. The cells were analysed in both soluble and insoluble forms to assess the nature of recombinant protein produced. Briefly, for soluble protein preparation cells were harvested at 4000 g for 20 min at 4°C. The pellets were dissolved in native lysis buffer containing lysozyme (1 mg/mL) and benzonase (nuclease inhibitor) (25 U/*μ*L), freeze-thawed at −80°C for three times, and centrifuged at 10,000 g for 30 min at 4°C to remove cell debris. Insoluble protein preparations were examined by lysing the cell pellets in denaturing lysis buffer for 45 min with agitation. Supernatants were separated using centrifugation. Recombinant proteins were purified by Ni-NTA Fast Start kit according to manufacturer's instruction (Qiagen). Protein concentrations were estimated using microtiter Bradford assay, using BSA as a standard [10]. 

### 2.4. SDS-PAGE and Western Blot Analysis

Thirty microlitres of purified sample was mixed with 30 *μ*L of sample loading dye and boiled for 5 min. Gel was stained with Coomassie brilliant blue, and the molecular weight of the protein was determined using standard molecular weight markers (Fermentas). Proteins were transferred to PVDF membrane (Millipore, India) from gel using an electrophoretic blotter (Bio-Rad). Nonspecific binding was blocked with blocking buffer containing 3% BSA. Dengue virus NS1 protein-specific monoclonal antibody 3D1.4 diluted in 1 : 750 in PBS/T buffer containing 2% milk powder which specifically recognizes linear epitope (kindly provided by A. K. Falconar, Universidad del Norte, Colombia) was used as a primary antibody. Blots were developed using ECL western blot kit (PIERCE).

### 2.5. RNA Extraction, RT-PCR, and Serological Assays

The RNA was extracted from 100 *μ*L of serum sample using QIAamp viral RNA mini kit (Qiagen). Detection of dengue virus has been done by RT-PCR according to methods described earlier [11]. Dengue IgM antibodies from serum samples were determined using dengue-IgM capture ELISA kit (NIV-Pune, India). 

### 2.6. Assessment of Biological Activity of Recombinant Antigen

Thioredoxin fusion tag was removed from the rNS1 using enterokinase (Invitrogen) according to the manufacturer's instruction. Assessment of biological activity (ability to detect dengue infection) was done with the known dengue positive and negative samples as well as with suspected samples collected during an outbreak investigation. A total of 56 suspected serum samples were screened. In order to avoid discrepancies, only primary dengue samples that were within the first 7 days after the onset of illness were tested. Samples were tested at the Centre for Research in Medical Entomology (WHO Collaborating Center for Lymphatic Filariasis and Dengue) Tamil Nadu, India. Solid phase ELISA was carried out in an overnight antigen coated microtiter plate. Briefly, recombinant antigen at a concentration of 15 *μ*g/mL was diluted in carbonate-bicarbonate buffer, coated in a microtiter plate, and kept overnight at 4°C after which, the wells were postcoated with NS1 monoclonal antibody diluted in 1% gelatin in PBS for 1.5 h at 37°C. Positive and negative human serum samples were diluted in 1 : 100, added in the wells, and kept at 4°C overnight. After washing with PBS for 5 times, the unbound complex was removed. Mouse anti-human IgG conjugated with HRP (Sigma) was added to the wells and incubated at 37°C for 1.5 h. After six washes with PBS, urea peroxidase (Sigma) and ortho-phenylenediamine dihydrochloride (Sigma) were added as substrates, and the plates were allowed to develop color at dark for 20 min. After the development of color, reaction was stopped with 1N H_2_SO_4_, and the OD was measured at 492 nm. The cutoff values were identified using the negative controls provided in the kit (using the formula, 4X negative control). A total of three negative controls were tested, and mean and standard deviations were calculated and used as cutoff value.

## 3. Results

### 3.1. Construction of Recombinant Plasmid and Clone Expressing NS1

**Figure 1:**
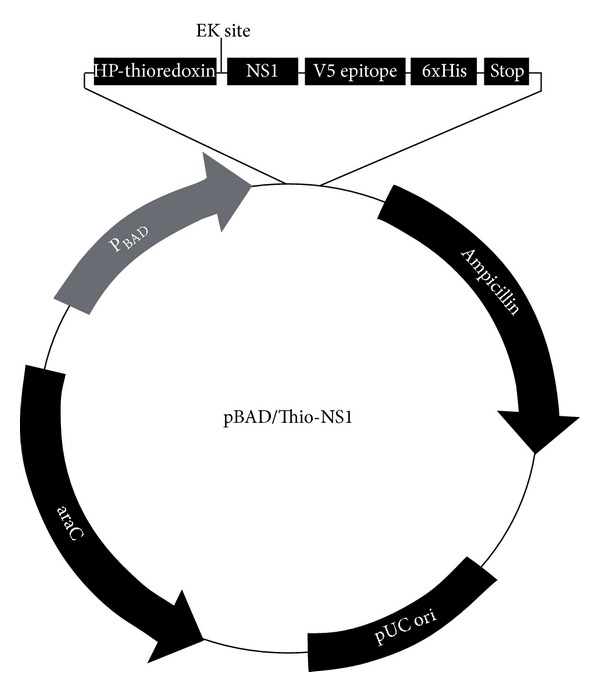
Recombinant plasmid showing the cloned NS1 gene between enterokinase (EK) site (for cleaving thioredoxin fusion protein) and his-tag (for easier purification).

Recombinant plasmid pBAD/Thio-TOPO NS1 was constructed using the amplified NS1 gene. The amplified NS1 gene was found to be 1.1 kb in size. The gene was ligated in such a way that the thioredoxin tag was fused upstream while histidine tag was in downstream position. The enterokinase cleavage size is present just upstream of the cloned NS1 gene (Figure 1). The cloned gene in the recombinant plasmid was verified by sequencing and compared. 

### 3.2. Recombinant Protein Production and Purification

**Figure 2:**
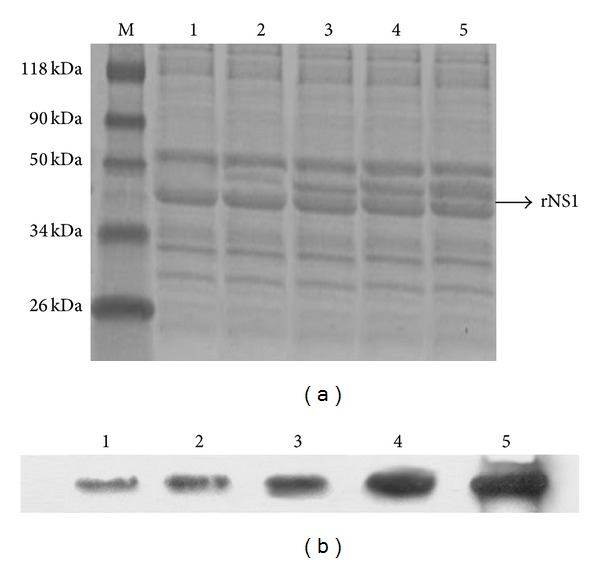
SDS-PAGE analysis of protein extracted in different time intervals from *E. coli *cells after being transformed with plasmid pBAD/Thio-NS1 and induced with 0.2% arabinose at 30°C. Lane M: protein marker and Lanes 1–4: protein extracts from different time intervals of 1, 2, 3, 4, and 5 h, respectively (a). The expressed protein in *E. coli *(after the EK cleavage) has been recognized by mouse monoclonal antibodies raised against dengue NS1 in western blot (b).

**Figure 3:**
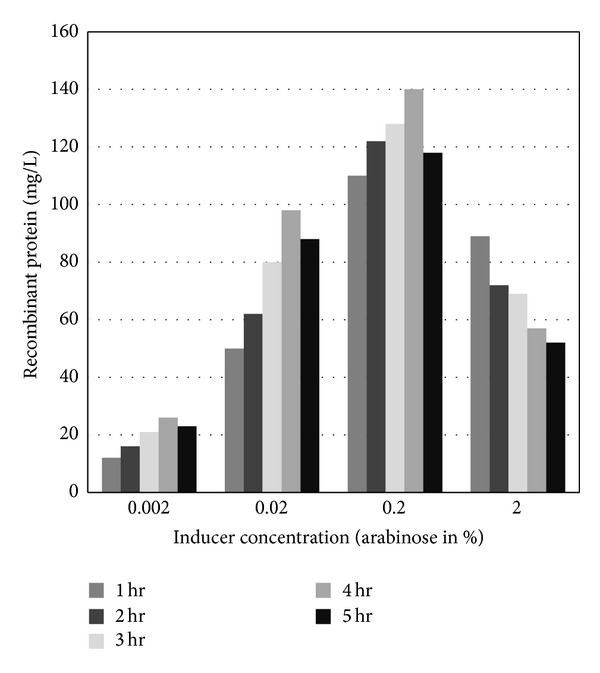
Recombinant NS1 antigen production at 30°C with different concentrations of arabinose.

Confirmed colonies containing the cloned NS1 gene (verified by colony PCR using insert- and sequence-specific primer; plasmid isolation and restriction mapping) were induced with different concentrations of arabinose (0.002%, 0.02%, 0.2%, and 2%) at 27°C, 30°C, and 35°C. Crude protein extracted was treated under native and denaturing conditions and purified by his-tagged column. The purified proteins were analyzed in SDS-PAGE, and proteins were found mostly in soluble form (data not shown) and subjected to western blot. In SDS-PAGE, the protein was spotted around 50 kDa (Figure 2(a)). The molecular weight of rNS1 includes additional 3 kDa that represents histidine tag. Thus the original molecular weight of the rNS1 was 47 kDa. Maximum protein of 140 mg/L was obtained after 4 hrs at 30°C with 0.2% arabinose induction (Figure 3).

### 3.3. Fine Tuning of Arabinose Induction with Glucose

**Figure 4:**
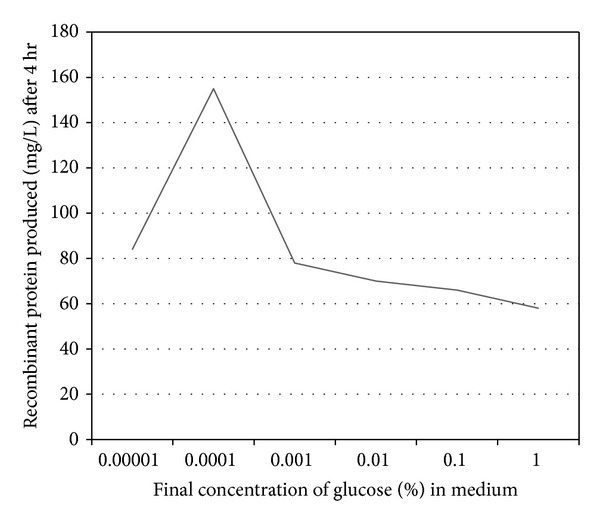
Effect of repressor on total protein produced. Recombinant proteins were induced with 0.2% arabinose and subsequently added with different concentrations of glucose. Proteins were extracted after each hour, purified, and quantified in microtiter Bradford assay.

Generally arabinose induction by pBAD is repressed in presence of glucose. To find whether addition of small amount of repressor (glucose) has an impact on the expression of recombinant protein, varying amounts of glucose to final concentrations of 0.00001%, 0.0001%, 0.001%, 0.01%, 0.1%, and 1% were added to the medium containing 0.2% arabinose. Presence of glucose at a final concentration of 0.0001% increases the amount of protein produced to 155 mg/L without affecting the quality of the protein produced. Increasing or decreasing the final concentration of glucose above or below 0.0001% does not increase total protein level (Figure 4). 

### 3.4. Confirmation of Recombinant Protein

Recombinant proteins separated on SDS-PAGE were transferred to PVDF membrane and blotted with anti-NS1 monoclonal antibody 3D1.4 recognizing the linear epitope (LX1 113-YSWKTWG-119) on NS1 protein (Figure 2(b)) [12]. The recombinant NS1 tagged with histidine tag was also confirmed by blotting with anti-histidine monoclonal antibody (data not shown). 

### 3.5. Solid Phase ELISA

**Figure 5:**
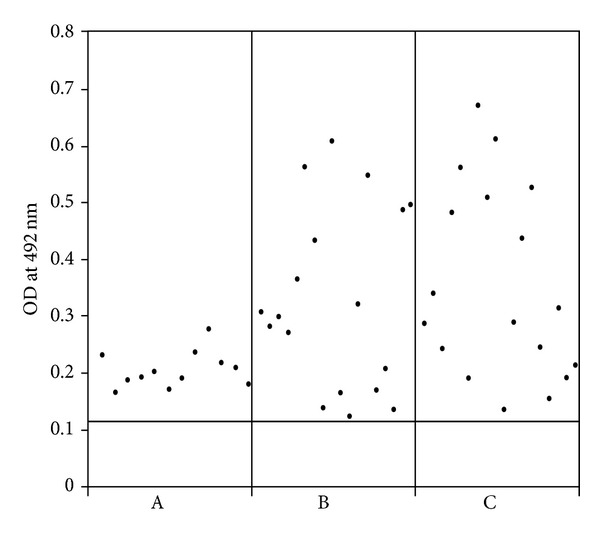
Comparison of recombinant NS1 protein in diagnosing acute dengue infection. A: RT-PCR positive, B: MAC-ELISA positive, and C: NS1 IgM ELISA positive.

The functional and immunological properties of recombinantly expressed NS1 antigen were tested in antibody detection ELISA. Purified NS1 antigen was coated in ELISA plates and tested for its ability to detect dengue virus NS1 specific antibody from clinical samples. To determine the cutoff value, fifty negative control samples were used (Figure 5). The clinical samples were collected during a suspected dengue outbreak. Routinely used diagnostic methods like RT-PCR MAC-ELISA were done alongside. The clinical samples tested were collected within 0–6 days after the onset of illness. Among the tested samples, RT-PCR detects 12 positives, while MAC-ELISA and NS1 IgM ELISA detect 18 positives, and all the MAC-ELISA positive samples were detected positively by NS1 IgM ELISA. 

## 4. Discussion 

The choice of using an expression system depends on factors including the intended use of the protein to be produced, the biological nature of the protein, quantity of required protein, and cost and time factors involved in production and purification. Most recombinant proteins are not efficiently produced in *E. coli* due to differences in codon biases, toxicity, mRNA instability, and lack of posttranslational modification [13, 14]. Researchers usually employ the application of refolding solutions to attain native protein after purification [15, 16]. The accumulation of recombinant protein as insoluble aggregates (inclusion bodies) is the most common problem associated with heterologous protein production in *E. coli*. These aggregated proteins are misfolded and biologically inactive [17]. 

Dengue NS1 protein contains two conserved N-glycosylation sites and 12 invariant cysteine residues forming six disulfide bonds [18, 19] which are essential for its biological function. Earlier, disulfide bond was induced *in vitro* during refolding step. Presence of six disulfide bonds in native NS1 protein may give rise to insoluble protein formation when produced heterologously. There are several reports of cloning and expression of dengue NS1 protein for research and diagnostic purpose in a variety of expression systems like bacteria, yeast, and insects. Among various systems available for the recombinant protein expression in *E. coli*, we have chosen pBAD promoter-based system for tightly controlled high-level expression. The vector has pUC origin of replication, optimal ribosome binding site, and his-patch thioredoxin fusion for efficient translation and purification. 

Production of rNS1 was at the center stage for most of the years as many researchers reported high yields of protein by using different expression systems and purification methods. In *E. coli*, Huang and his coworkers reported yield of 10–30 mg/L protein [20], Lazaro-Olán and his colleagues had reported 8–12 mg/L [21], and 230–250 mg/L was reported by Das and his coworkers [22]. Recently, 135 mg/L was obtained by experiments conducted by Amorim and his coworkers [23]. Zhou and his coworkers had successfully expressed NS1 in *Pichia pastoris* and obtained 70 mg/L [24]. Though the amount of rNS1 produced here (155 mg/L) is lesser than what Das and his coworkers reported earlier, their experiment is time consuming and costly as they optimized codon and employed refolding conditions to attain protein in native form. 

Addition of agent like GSH/GSSG during refolding increases soluble protein production by maintaining oxidized environment and enhances disulfide bond formation [25, 26]. Previously, researchers found that thioredoxin can accumulate up to 40% of total protein content and remains soluble when overexpressed in *E. coli* [27]. Thioredoxin was shown to act as a chaperone during protein folding [28, 29] and found to hold the folding intermediates of the heterologously expressing protein in soluble form until the protein attains its final conformation [27].

Soluble protein production can be obtained by a variety of techniques reviewed by the authors in [9]. Usually solubility is increased by tuning down production rate, decreasing the strength of promoter activity, and increasing molecular chaperons. The araC gene of pBAD promoter acts as both positive and negative regulator [30] and can be repressed in presence of glucose [31]. In a novel approach, we added different concentrations of glucose (repressor) to the medium and analysed its impact on recombinant protein produced. Surprisingly, glucose at 0.0001% concentration at 30°C in 0.2% arabinose increases the total quantity of protein produced from 140 mg/L to 155 mg/L (Figures 3 and 4). We hypothesize that the enhancement seems to be solely dependant on equilibrium between the repressor and inducer concentrations. Increase in inducer and/or repressor concentration does not increase the quantity of recombinant protein produced; rather it shifts equilibrium and results in the inclusion body formation. 

For the evaluation purpose, we chose clinical samples collected between 0 and 6 days after the onset of illness, as there will be no detectable amount of IgM in serum before 3 days and viral load in serum starts to decrease after day 6. Our results demonstrated that rNS1 tagged with histidine patch thioredoxin has been able to retain its biological activity (in ELISA), and the protein was recognized by monoclonal antibody (specifically recognizing a linear epitope). Fusion proteins (like his-patch thioredoxin) have been shown to be highly soluble and fold efficiently. The purified rNS1 protein has been shown to retain its antigenicity and functional properties. Kappa statistics demonstrated substantial agreement between the RT-PCR test and the NS1 IgM ELISA (*κ*  value = 0.73), and it is almost perfect between the MAC-ELISA and the NS1 IgM ELISA (*κ*  value = 1).

In conclusion, high level production of soluble recombinant dengue-2 NS1 can be produced from *E. coli* by choosing appropriate vector containing suitable fusion partners, promoter (tightly control and regulatable), and other production strategies like optimization of temperature. A similar kind of approaches can be used for the production of other important viral proteins for research and diagnostic purposes.
